# Beyond a Biomarker: Investigations of a Proinflammatory Role for Cell-Free DNA in Liver Transplant Ischemia and Reperfusion

**DOI:** 10.3389/ti.2026.15340

**Published:** 2026-04-08

**Authors:** Mike Schnepppfister, Yue Wang, Chen Zhong, Tori Huey, Hesham El-Shewy, Yichu Kao, Joseph R. Scalea, Thomas A. Morinelli, Yuan Zhai, Dirk J. van der Windt

**Affiliations:** The Lee Patterson Allen Transplant Immunobiology Laboratory, Department of Surgery, Medical University of South Carolina, Charleston, SC, United States

**Keywords:** cell free DNA, inflammatory cytokines, ischemia and reperfusion, liver transplantation, macrophages

## Abstract

Donor-derived cell-free DNA (dd-cfDNA) is a biomarker for rejection after organ transplantation. We hypothesized that high release of cfDNA immediately after liver transplant also has a biologic role in inflammation in ischemia and reperfusion injury (IRI). To investigate this concept, C57BL/6 mice were subjected to 90 min *in situ* liver ischemia. After 6 h reperfusion, cfDNA was purified from serum and used to stimulate macrophages *in vitro*, which resulted in production of high levels of inflammatory cytokines TNFα and IL-6, and chemokine CXCL10. Enzymatic degradation of cfDNA by DNase I inhibited these inflammatory responses (e.g., TNFα: DNase I 48.1 ± 37.4 vs. untreated 1,030 ± 206 pg/mL, p = 0.0001). cfDNA from netosis-deficient PAD4KO mice was found to be equally pro-inflammatory compared to wild type cfDNA (TNFα: PAD4KO 1048 ± 199 vs. wild-type 1,162 ± 150 pg/mL, p = 0.64), indicating its mechanism is not dependent on neutrophils undergoing netosis. Next, a single dose of DNase I was added to the perfusate during rat liver normothermic machine perfusion (NMP) to significantly reduce perfusate cfDNA levels (384 ± 132 to 129 ± 18 ng/mL, p = 0.026). In conclusion, our data suggest that cfDNA can have pro-inflammatory effects during liver IRI beyond being a biomarker. DNase I may be a promising therapeutic intervention during NMP to reduce the graft’s inflammatory propensity prior to implantation.

## Graphical Abstract

**Figure:**
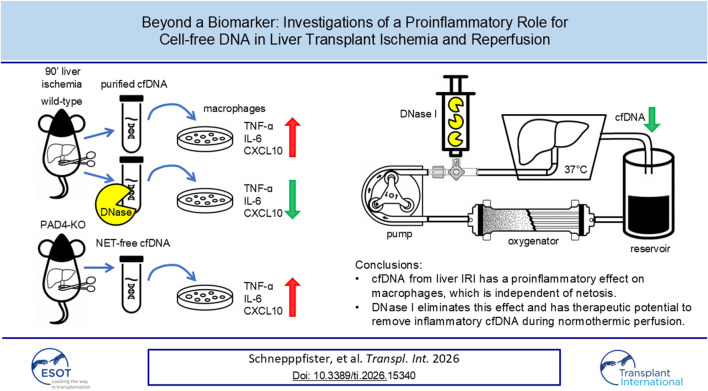

## Introduction

In organ transplantation, circulating donor-derived cell-free DNA (dd-cfDNA) has been established as a reliable biomarker for rejection, and “liquid biopsy” blood tests have been developed for screening and early detection [1]. In liver transplantation, dd-cfDNA is also associated with non-rejection liver graft cellular injury [2]. The greatest insult causing cellular injury occurs during ischemia and reperfusion at the time of liver implantation: at this time, cfDNA levels are more than 10 times higher than in controls [3] and 80%–90% of cfDNA is donor-derived (compared to ∼30% during a biopsy proven rejection episode) [4]. It is still largely unknown if cfDNA has biologic activity in the pathogenesis of organ graft injury. In other inflammatory conditions however, extracellular DNA has been investigated as a damage-associated molecular pattern (DAMP) that can activate innate immune cells to initiate and/or propagate the sterile inflammatory response [5, 6].

Ischemia and reperfusion injury (IRI) is inherent to liver donation and transplantation [7]. While a young, healthy liver graft can readily recover from IRI, marginal liver grafts have a lower tolerance for IRI. After transplantation of a marginal liver, IRI can result in reperfusion syndrome, early allograft dysfunction, acute kidney injury, and prolong a recipient’s hospital stay and recovery [8]. Because of organ donor shortage, we depend on the use of marginal donors for liver transplantation (older, having fatty liver, donation after circulatory death). Therefore, understanding the mechanisms of IRI remains important and elucidating a bioactive role for cfDNA can be of significance in developing treatment strategies to inhibit IRI.

Here we investigated the hypothesis that liver-derived cfDNA has a pro-inflammatory role beyond a biomarker in ischemia-reperfusion injury. We also explored if DNase I can be used to neutralize pro-inflammatory cfDNA, and whether this could be applied during NMP to clean the liver from cfDNA even prior to implantation.

## Methods

### Animals and Surgical Procedures

Animal protocols were in adherence with the NIH Guide for the Care and Use of Laboratory Animals, and were approved by the Institutional Animal Care and Use Committee. Details on mouse strains and surgical procedures are available in the Supplementary Methods. In mice, 90 min of *in situ* partial warm-ischemia to the liver was applied [9]. Reperfusion was initiated, and mice were euthanized at various time points for serum collection. The rat donor hepatectomy procedure included laparotomy, hepatic artery ligation, bile duct and portal vein cannulation, and *in situ* liver flush with cold heparinized PBS until the effluent from the vena cava appeared clear [10].

### Cell-Free DNA Purification

cfDNA was purified from mouse serum using the QIAamp Circulating Nucleic Acid Kit, Qiagen, Hilden, Germany, to exclude other pro-inflammatory stimuli such as cytokines and chemokines. cfDNA was quantified using Quant-iT PicoGreen dsDNA Assay Kit, ThermoFisher Scientific, Waltham, MA. DNase I, Millipore Sigma, St. Louis, MO, was added to purified cfDNA at escalating concentrations of 0.1, 0.5, 1, and 5 mg/mL and incubated at 37 °C for 30 min, followed by measurement of remaining cfDNA concentrations.

### Macrophage Culture and Stimulation

RAW 264.7 cells (ATCC, Manassas, VA) were seeded onto 96-well plates at 75,000 cells/well and incubated overnight at 37 °C. Peritoneal macrophages were collected from C57BL/6J mice, and cultured as previously described [11], and as detailed in the Supplementary Methods. Macrophages cultures were washed once and incubated with medium containing 5000 ng/mL purified cfDNA. cfDNA was complexed with lipofectamine for intracellular delivery as was previously suggested by Kaczorowski et al. [12] cfDNA from wild type mice was compared to DNase I-treated cfDNA, and cfDNA from PAD4KO mice. After 24 h stimulation, supernatants were analyzed for TNF-α, IL6, and CXCL10 using ELISA (ThermoFisher Scientific).

### Normothermic Machine Perfusion

A custom-designed NMP system for rat livers was developed together with Harvard Apparatus, Holliston, MA. Livers were perfused via the cannulated portal vein under controlled conditions for oxygenation, flow, and pressure at temperature of 37 °C. Details on the perfusate composition are provided in the Supplementary Methods. Perfusate samples were collected every 15 min. After 120 min, DNase I was added to the perfusate at a concentration of 0.5 mg/mL.

### Statistical Analysis

Continuous variables were expressed as mean ± SD or mean ± SEM. A two-tailed independent t-test was used to compare experimental groups. Repeated measures ANOVA was used to compare cfDNA values during NMP. All analyses were performed with GraphPad Prism and a P value < 0.05 was considered statistically significant.

## Results

### Liver Ischemia Causes High Levels of Circulating cfDNA

In our experience, 90 min of portal clamping results in significant IRI without being lethal [9]. All mice survived the operation and returned to normal activity upon emergence from anesthesia. Quantification of cfDNA in serum revealed that cfDNA levels steeply rise upon reperfusion, peak at 6 h post-reperfusion (15,594 ± 3,728 vs. 1,048 ± 289 ng/mL after sham laparotomy, n = 6, p < 0.0001), and return to baseline levels by 24 h (Supplementary Figure S1). A similar pattern has been observed in liver transplant patients in whom cfDNA levels are highest at completion of the operation and have cleared 24 h postoperatively [3].

### Purified cfDNA has a Direct Pro-Inflammatory Effect on Macrophages

**FIGURE 1:**
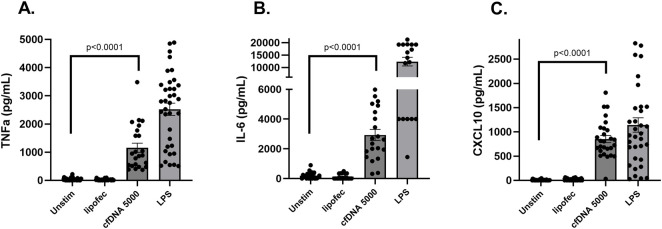
Inflammatory cytokine levels in mouse peritoneal macrophage cultures stimulated with purified cfDNA after liver ischemia and reperfusion. cfDNA-stimulated macrophages produced high levels of inflammatory cytokines TNF-α **(A)** and IL-6 **(B)**, and chemokine CXCL10 **(C)** compared to unstimulated and lipofectamine controls. Data represent the mean ± SEM of 5 or more independent experiments, each experiment performed in triplicate.

Purification of cfDNA from serum containing peak levels of cfDNA (6 h post-reperfusion) resulted in eluates containing 12,000–35,000 ng/mL cfDNA. Purified cfDNA was used to stimulate RAW cells and primary peritoneal macrophage cultures at 5000 ng/mL, a clinically relevant concentration observed in serum of patients undergoing liver transplant [3]. Supplementary Figure S2 represents our initial experiments where a release of TNF-α into the supernatant was observed upon stimulation of both RAW cells and peritoneal macrophages. This response was increased by complexing cfDNA with lipofectamine, which may indicate a role for an intracellular receptor mechanism [13]. In subsequent experiments, we therefore used cfDNA with lipofectamine to stimulate fresh peritoneal macrophage cultures. Macrophages were significantly activated to produce high levels of inflammatory cytokines TNF-α (1,155 ± 166 vs. unstimulated 36.8 ± 7.3 pg/mL, p < 0.0001) and IL-6 (2,932 ± 374 vs. unstimulated 190 ± 45 pg/mL, p < 0.0001) (Figures 1A,B). We investigated whether macrophages produced type I IFN-β [14]. IFN-β in supernatants was detected, however, the levels were variable (data not shown). As macrophages themselves express type I IFN receptors (IFNAR-1) [15], we hypothesized that IFN-β was partially being removed from supernatant by autocrine binding to INFAR-1. We therefore tested supernatants for CXCL10, a chemokine produced upon interferon pathway stimulation. Indeed, levels of CXCL10 were highly elevated (851 ± 74 vs. unstimulated 6.7 ± 1.7 pg/mL, p < 0.0001) (Figure 1C).

### DNase I Degrades cfDNA and Eliminates Its Inflammatory Effect

**FIGURE 2:**
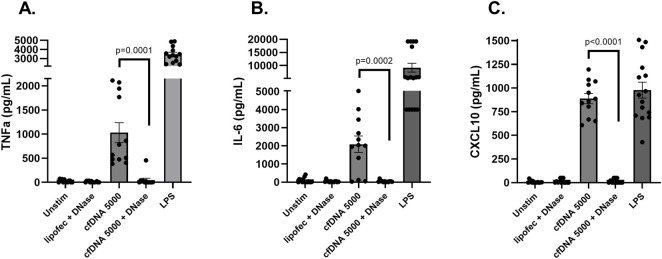
Cytokine production in mouse peritoneal macrophage cultures exposed to cfDNA that was pretreated with 5 mg/mL DNase I. DNase I-degraded cfDNA nearly completely lost its ability to induce the production of TNF-α **(A)**, IL-6 **(B)**, and CXCL10 **(C)** by macrophages. Data represent the mean ± SEM of 3 independent experiments, each experiment performed at least in triplicate.

Thus far, our results showed that circulating cfDNA resulting from liver IRI can have a pro-inflammatory effect on innate immune cells such as macrophages. As we previously used DNase I to limit IRI and steatohepatitis in mouse models [16, 17], we investigated the effect of DNase I in the *in vitro* macrophage stimulation assay. First, the effect of various concentrations of DNase I was tested and a dose-dependent degradation of cfDNA during 30min incubation at 37 °C was found (Supplementary Figure S3). We then used DNase I at 5 mg/mL concentration to pretreat cfDNA prior to macrophage stimulation. Figure 2 shows that cfDNA that had been digested by DNase I nearly completely lost its capacity to stimulate macrophages (TNFα: 48.1 ± 37.4 vs. untreated 1,030 ± 206 pg/mL, p = 0.0001; IL-6: 44.2 ± 15.5 vs. untreated 2090 ± 457 pg/mL, p = 0.0002; CXCL10: 14.4 ± 6.5 vs. untreated 888 ± 54 pg/mL, p < 0.0001), demonstrating again that cfDNA is inflammatory, and providing proof of concept that DNase I can be a potential treatment option to limit cfDNA-induced inflammation in liver transplant IRI.

### cfDNA Is Inflammatory Independent of Netosis

**FIGURE 3:**
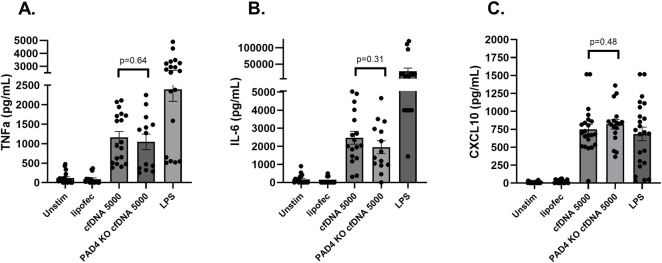
Comparison of cytokine production in mouse peritoneal macrophage cultures exposed to cfDNA purified from wild type vs. PAD4 KO mice. The production of TNF-α **(A)**, IL-6 **(B)**, and CXCL10 **(C)** induced by cfDNA from PAD4 KO mice was not significantly different than by cfDNA from wild type mice, indicating that inflammatory cfDNA is likely to originate from cell types other than the known inflammatory DNA released by neutrophils during netosis. Data represent the mean ± SEM of 4 independent experiments, each experiment performed at least in triplicate.

In previous work we focused on the inflammatory properties of extracellular DNA from neutrophils that form neutrophil extracellular traps (NETs) [17, 18]. Although it is known that netosis can release proinflammatory DNA into circulation [18], the very high levels of circulating cfDNA from liver IRI are unlikely the result of netosis alone. Thus, we tested whether cfDNA can induce inflammation independent of netosis. Liver IRI was applied to PAD4KO mice that are deficient in forming NETs. PAD4KO confers a degree of protection against IRI [18] but 90 min of liver ischemia still resulted in high levels of circulating cfDNA. Purified cfDNA from NET-free PADKO mice, used at the same concentration as wild type cfDNA, induced the production of TNF-α, IL-6, and CXCL10 to comparable levels (TNFα: PAD4KO 1048 ± 199 vs. wild-type 1,162 ± 150 pg/mL, p = 0.64; IL-6: PAD4KO 1952 ± 359 vs. wild-type 2,472 ± 354 pg/mL, p = 0.31; CXCL10: PAD4KO 820 ± 66 vs. wild-type 749 ± 69 pg/mL, p = 0.48) (Figure 3), indicating that the pro-inflammatory activity of cfDNA is independent of neutrophils undergoing netosis. To further investigate the composition of cfDNA, we applied PCR using specific primers for nuclear and mitochondrial DNA and found that liver IRI causes the release of both (details in Supplementary Methods and Supplementary Results).

### NMP as a Platform for *Ex Vivo* Treatment With DNase I

**FIGURE 4:**
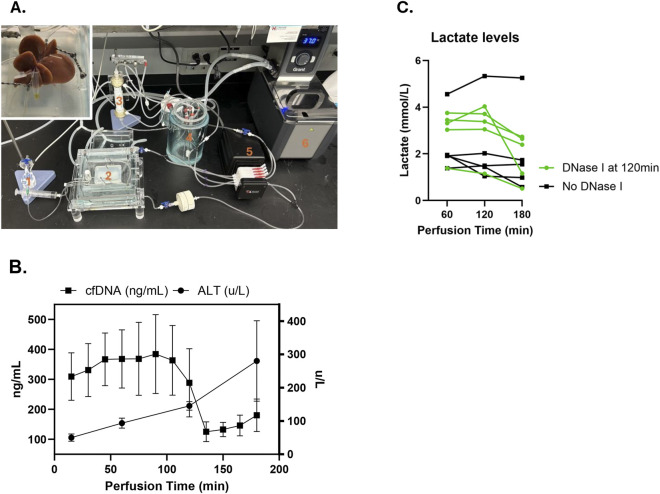
**(A)** Rat liver normothermic perfusion pump system. 1. Pressure transducer, 2. Organ chamber, 3. Oxygen membrane, 4. Perfusate reservoir, 5. Peristaltic pump, 6. Temperature-controlled water bath. Insert: rat liver during NMP with production of bile. **(B)** Perfusate ALT and cfDNA levels during rat liver NMP. cfDNA and ALT levels gradually increased over time. A single dose of DNase I for a concentration of 0.5 mg/mL administered at 120 min significantly reduced cfDNA levels (repeated measures ANOVA comparing cfDNA pre- and post-DNase I, p = 0.031). **(C)** Administration of DNase I did not affect ongoing clearance of lactate between 120 and 180 min (lactate data available for 5 independent experiments).

NMP offers a promising platform to deliver *ex vivo* treatment to the organ, and NMP at 37 °C would provide the optimal conditions for the enzymatic activity of DNase I. We established an NMP system for rat livers. Flows of 2 mL/g liver tissue were obtained while portal pressures remained low at 5–7 mmHg. Perfusate PaO_2_ was kept >80 mmHg and livers produced bile while being perfused (Figure 4A). During NMP, ALT levels gradually increased over time, comparable to the pattern of ALT release in our clinical liver NMP program. Adding a single dose of DNase I to the perfusate for a concentration of 0.5 mg/mL (10x lower than used in our *in vitro* experiment) resulted in a reduction of perfusate cfDNA levels from 384 ± 132 to 129 ± 18 ng/mL, indicating that DNase I has activity in the currently used NMP setup. Repeated measures ANOVA demonstrated that post-DNase I cfDNA levels were significantly lower than pre-DNase I levels, p = 0.031 (Figure 4B). The performance of the liver during NMP appeared unaffected by DNase I addition. While flow was kept consistent, no difference in portal vein pressure was noted (5.77 ± 0.51 mmHg pre-DNase I vs. 5.69 ± 0.41 mmHg post-DNase I, p = 0.92). After DNase I administration at 120min, lactate clearance continued for all livers, in a pattern that did not appear different from liver perfusions during which DNase I was not applied (Figure 4C). Although not quantified, bile production was not altered by DNase I.

## Discussion

Donor-derived cell-free DNA (dd-cfDNA) has diagnostic value as a biomarker for early detection of organ rejection after transplantation. Here we investigated a biologic activity of cfDNA beyond being a biomarker. We found that liver-derived cfDNA purified from serum after liver ischemia and reperfusion is a direct pro-inflammatory stimulus for macrophages. DNase I effectively degraded cfDNA and eliminated its pro-inflammatory effect *in vitro*. Applying DNase I during liver NMP reduced cfDNA levels in the perfusate, suggesting that addition of DNase I during NMP can be further explored as a strategy to clean the liver graft from inflammatory cfDNA even prior to transplantation.

After reperfusion of a liver, serum dd-cfDNA levels are multiple times higher than levels that would indicate rejection [4]. With our mouse model of *in situ* liver IRI, we confirmed that ischemia-reperfusion is indeed the responsible phenomenon for this massive release of cfDNA. Excessive cfDNA release can overwhelm natural serum DNases and result in inflammatory immune stimulation. In trauma and severe sepsis for example, the levels of serum cfDNA correlate with higher levels of inflammation and worse clinical outcomes [19, 20]. Under those conditions, cfDNA may act as a DAMP that activates pattern recognition receptors on innate immune cells. Conversely, when a genetic mutation results in DNase deficiency, low levels of cfDNA from physiologic cell turnover lead to autoimmune disease (systemic lupus erythematosus) in humans and mice, and immune activation leads to the presence of anti-DNA antibodies [21, 22]. Although the significance of DAMPs in transplant immune responses has been recognized [23, 24], a biologic activity for cfDNA in liver IRI has not been established, and to our knowledge, there have also been no investigations of targeting cfDNA to reduce IRI. While we have yet to confirm this in *in vivo* models of liver transplant, our data provide the initial mechanistic proof of this concept.

DNase I has previously been used for its anti-inflammatory effects in rodent models of IRI [16, 25], and steatohepatitis [17]. We have previously shown that treatment with exogenous DNase I can alleviate NET-induced inflammation [16, 17]. In liver transplantation, the high levels of circulating cfDNA after reperfusion are unlikely the result of netosis alone, and we hypothesized that cfDNA can be inflammatory independent of netosis. NET-free cfDNA from PAD4KO serum retained its capability of activating macrophages, indicating that cfDNA from other cell types and other mechanisms than netosis can be pro-inflammatory. Using PCR we found that cfDNA contains both nuclear and mitochondrial DNA; defining the specific role of each in liver IRI will be important, as mitochondrial DNA has been shown to be a DAMP in transplant inflammation [26].

Ideally, one would limit the liver graft DAMP-burden even prior to organ implantation. NMP offers a promising yet greatly underutilized platform to deliver *ex vivo* treatment, and normothermia provides the optimum conditions for DNase, an enzyme active at body temperature. We found that the addition of DNase I to the perfusate greatly reduced cfDNA levels in a rat liver NMP model, without affecting pump parameters and lactate levels. If this were to be developed toward a clinical application, DNase I seems to have a favorable safety profile. Patients with lupus nephritis have been safely treated with recombinant human DNase [27]. Moreover, *ex vivo* use during NMP would avoid direct exposure of the recipient patient.

Overall, we found that cfDNA from liver IR can be more than a biomarker for cellular injury. We acknowledge that our results thus far are based on *in vitro* and *ex vivo* observations and that there are several remaining questions to be answered. First, we next need to perform experiments of liver reperfusion and transplantation after DNase I treatment to understand the clinical benefit of targeting cfDNA for therapeutic purposes. Second, we acknowledge that our studies have used peritoneal macrophages as target cells, as they can be consistently obtained and cultured. We have not yet been able to establish Kupffer cell cultures. We believe that our model is relevant since both peritoneal macrophages and Kupffer cells are tissue resident macrophages derived from common yolk sac progenitors [28]. However, it will be important to define the specific responses to cfDNA of Kupffer cells versus infiltrating macrophages, as the roles of macrophage subtypes in response to inflammation and resolution are increasingly becoming understood [29, 30]. Third, we need to investigate the signaling mechanisms in further detail. Although lipofectamine was not required to elicit a cytokine response, creation of a lipid bilayer (liposome) for intracellular delivery of cfDNA increased the pro-inflammatory response. Several known intracellular nucleotide receptors exist and include Toll-like receptor 9 (TLR9), stimulator of interferon gene (STING), and absent in melanoma 2 (AIM2). These receptors all have been implied in liver IRI [31–33], but their exact response to cfDNA has yet to be determined. *In vivo* equivalents of liposomes to fuse with the macrophage membrane could include DNA packaged in extracellular vesicles for which a role has been suggested [34], but not yet confirmed.

In conclusion, here we show that cfDNA has a pathogenic role beyond being a marker of graft injury. cfDNA resulting from liver IRI was directly inflammatory in macrophage cultures and DNase-digestion greatly eliminated this effect. It was applied during NMP to actively reduce the level of cfDNA. Understanding the pathophysiologic role of cfDNA has significance in the development of treatment strategies to inhibit IRI, and to explore the tremendous potential for *ex vivo* treatment during liver NMP.

## Data Availability

The raw data supporting the conclusions of this article will be made available by the authors, without undue reservation.
