# Supplementary material for: Beyond a Biomarker: Investigations of a Proinflammatory Role for Cell-Free DNA in Liver Transplant Ischemia and Reperfusion
Source: Transpl Int. 2026 Apr 8;39:15340. doi: 10.3389/ti.2026.15340 (PMC13099445; doi:10.3389/ti.2026.15340)

**Supplementary Materials**

**Supplementary Methods**

**Animals**

Wild type C57BL/6J mice were bred in our laboratory. B6.Cg-Padi4tm1.1Kmow/J  (PAD4KO) mice were purchased from Jackson Laboratories, Bar Harbor, ME. Sprague-Dawley rats weighing 300-500g were purchased from Charles River Laboratories, Worcester, MA.

**Surgical procedures**

*Mouse liver partial warm-ischemia model.* Female and male mice were anesthetized with 2-5% isoflurane and placed on 37°C heating pad. Heparin 2.5u was injected via tail vein, and carprofen 5ug/g body weight was administered for perioperative analgesia. After midline laparotomy, an atraumatic clip was used to interrupt arterial and portal venous blood flow to the cephalad liver lobes for 90 minutes.^1^ The clip was removed to initiate reperfusion. Sham controls underwent laparotomy without vascular occlusion. Mice were sacrificed at various time points and serum samples were collected.

*Rat donor hepatectomy.* Hepatectomies were performed on both sexes of rats as previously described.^2^ In short, laparotomy was performed and the hepatic artery was ligated. The bile duct was cannulated with 30G angiocatheter. The portal vein was cannulated, and the liver was flushed with 40 mL cold heparinized PBS until the effluent from the vena cava appeared clear. The suprahepatic and infrahepatic vena and perihepatic ligaments were divided and the liver was removed from the body.

**Peritoneal macrophage culture and stimulation**

Peritoneal macrophages were collected from C57BL/6J mice as previously described.^3^ Briefly, mice were injected with 5mL of DMEM into the peritoneal cavity and fluid was collected from the abdomen. The cell-containing medium was centrifuged at 1200 rpm at 4°C for 6 minutes, the supernatant discarded, and the cells treated with 2 mL RBC lysis buffer for 2 minutes. After two washings, the cells were reconstituted in DMEM +10% FBS and 250,000cells/well were seeded into 96-well plates and incubated for 1.5-2hrs at 37°C, 5% CO2. Media in the wells was then aspirated to remove nonadherent/non-macrophage cells and 200ul of DMEM 10% FBS added back. We have previously established that this method results in macrophage cultures with >90% purity as determined by flow cytometric analysis for CD11b and F4/80.^4^

Macrophages cultures were washed once and incubated with medium containing 5000ng/mL purified cfDNA. LPS at 200ng/mL was used as positive control. In all experiments, macrophages were stimulated for 24h at 37°C, 5% CO2, and supernatants were collected, spun and stored at −80°C. Supernatants were analyzed for TNF-α, IL6, and CXCL10 using ELISA (ThermoFisher Scientific). Alanine aminotransferase levels (ALT) in NMP perfusates were quantified using Pointe Scientific ALT (SGPT) Liquid Reagents assay, Fisher Scientific.

**PCR to determine a nuclear vs. mitochondrial origin of cfDNA**

To determine the proportion of mitochondrial versus nuclear DNA, we performed SYBR Green-based quantitative PCR (qPCR) on purified cfDNA from mouse serum after liver ischemia and reperfusion. The primers were specific for mitochondrial (gene ND4), and nuclear DNA (B2M) which was designed free from exon junctions to amplify non-spliced nuclear genome sequences. qPCR conditions were as follows: (preamplification step) 95°C for 5min; (amplification step) 45 cycles of 95°C for 10s, 60°C for 10s, and 72°C for 20s; and (melting curve) 66–97.5°C in 0.5°C increments. The number of ND4 copies relative to nuclear copies (=2 for diploid cells) was determined using the following equations: ^5^

ΔCt = Ct(B2M) – Ct(ND4)

mtDNA copy number = 2x2ΔCt.

The proportion of mtDNA was then calculated as follows: ^6^

_ (mtDNA copies) × (mtDNA genome size) _

(mtDNA copies × mtDNA genome size) + (2 × nuclear genome size)

**NMP perfusate composition and setup**

The perfusate consisted of Williams E medium supplemented with BSA (12.5%), Gibco Glutamax (0.672%), 0.0013 units/ml insulin, 0.0077 mg/ml hydrocortisone, 0.01 units/ml heparin, 76.7 units/ml penicillin, and 76.7 units/ml streptomycin. No red blood cells or oxygen carrier were added. The perfusate was oxygenated by an in-line positioned Type D150 membrane oxygenator to achieve PaO_2_ >80mmHg (usually >200mmHg is readily achieved). Flows of 2mL/g liver tissue were established while portal vein pressures remained low at 5-7mmHg. Temperature of 37°C was maintained by the water jacketed system, and verified directly on the liver surface using an infrared thermometer. The perfusate pH was adjusted to 7.4 using sodium bicarbonate and verified by iSTAT gas analysis.

**Supplementary Results**

**Nuclear vs. mitochondrial origin of cfDNA**

qPCR was performed in triplicates resulting in close-ranged Ct values for nuclear B2M (19.66±0.15) and mitochondrial ND4 (17.27±0.059). By the above equation, there were 10.5x more copies of mtDNA than nDNA in the cfDNA purified from mouse serum after liver ischemia and reperfusion. Because of the marked difference in size of the mitochondrial vs. nuclear genome (1.66*10^4^ bp vs. 2.6*10^9^ bp ), the proportion of mtDNA relative to the total mass of cfDNA remains small at 0.00335%, although this is under the assumption that the entire nuclear genome leaked from necrotic cells.

**References**

1. Zhang H, Ni M, Wang H, et al. Gsk3β regulates the resolution of liver ischemia/reperfusion injury via MerTK. JCI Insight. Jan 10 2023;8(1) doi:10.1172/jci.insight.151819

2. Beal EW, Dumond C, Kim JL, et al. A Small Animal Model of Ex Vivo Normothermic Liver Perfusion. J Vis Exp. Jun 27 2018;(136) doi:10.3791/57541

3. Ray A, Dittel BN. Isolation of mouse peritoneal cavity cells. J Vis Exp. Jan 28 2010;(35) doi:10.3791/1488

4. Gonçalves R, Mosser DM. The Isolation and Characterization of Murine Macrophages. Current Protocols in Immunology. 2015;111(1):14.1.1-14.1.16. doi:https://doi.org/10.1002/0471142735.im1401s111

5. Quiros PM, Goyal A, Jha P, Auwerx J. Analysis of mtDNA/nDNA Ratio in Mice.

Curr Protoc Mouse Biol. 2017 Mar 2;7(1):47-54. doi: 10.1002/cpmo.21.

6. Chu HT, Hsiao WW, Tsao TT, et al. Quantitative assessment of mitochondrial DNA copies from whole genome sequencing. BMC Genomics. 2012;13 Suppl 7(Suppl 7):S5. doi: 10.1186/1471-2164-13-S7-S5.

**Supplementary Figure Legends**

**Supplementary Figure 1**. cfDNA levels in mouse serum following liver ischemia for 90min and reperfusion for various times. cfDNA was released from the liver to highly elevated levels compared to sham-operated controls, peaked at 6h and returned to baseline levels by 24h post-reperfusion. Data represent n>6 mice for timepoints 6, 8 and 24h, n=3 mice for the remaining timepoints.

**Supplementary Figure 2**. Preliminary experiments on TNF-α production in RAW 264.7 cells and in peritoneal macrophages. In both cell cultures TNF-α was released into supernatant in response to stimulation with purified cfDNA. The addition of lipofectamine was not required to induce this response, but it increased TNF-α production, which may indicate a role for an intracellular receptor mechanism.

**Supplementary Figure 3**. cfDNA degradation during incubation with varying concentrations of DNase I for 30min at 37ºC. The degradation of cfDNA was dose-dependent, with near complete degradation observed at 5 mg/mL DNase I. Data represent the mean±SD of 3 independent experiments.


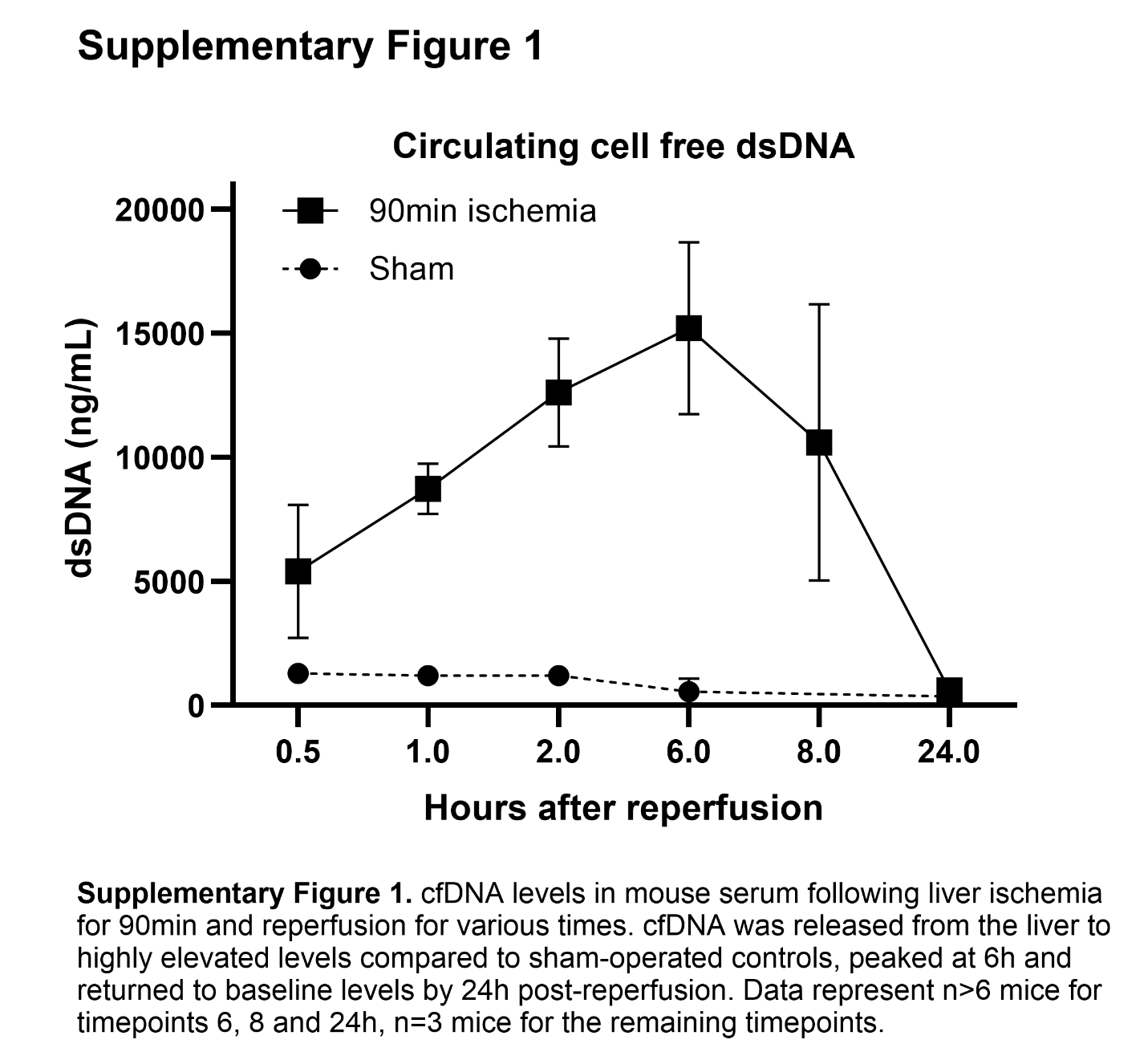


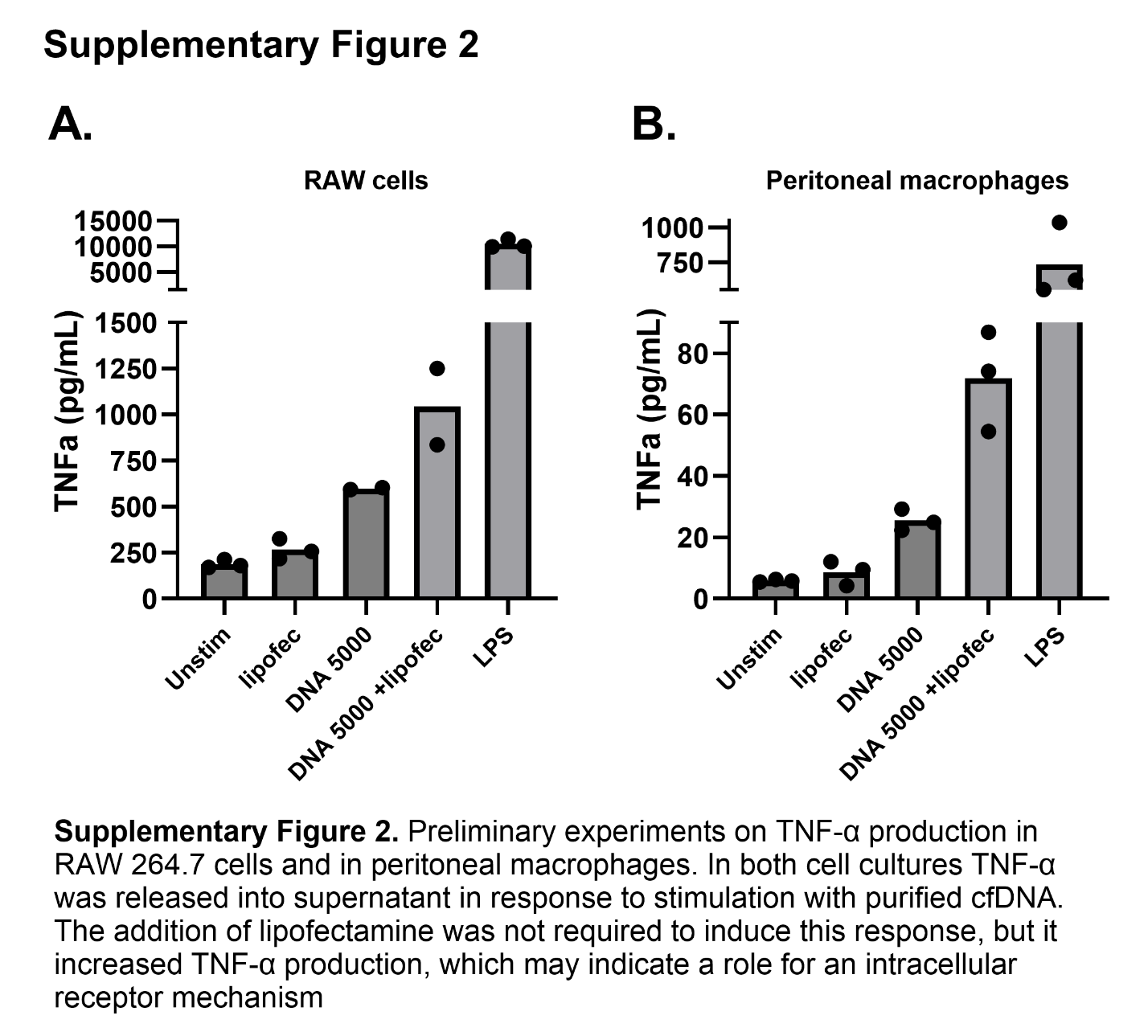


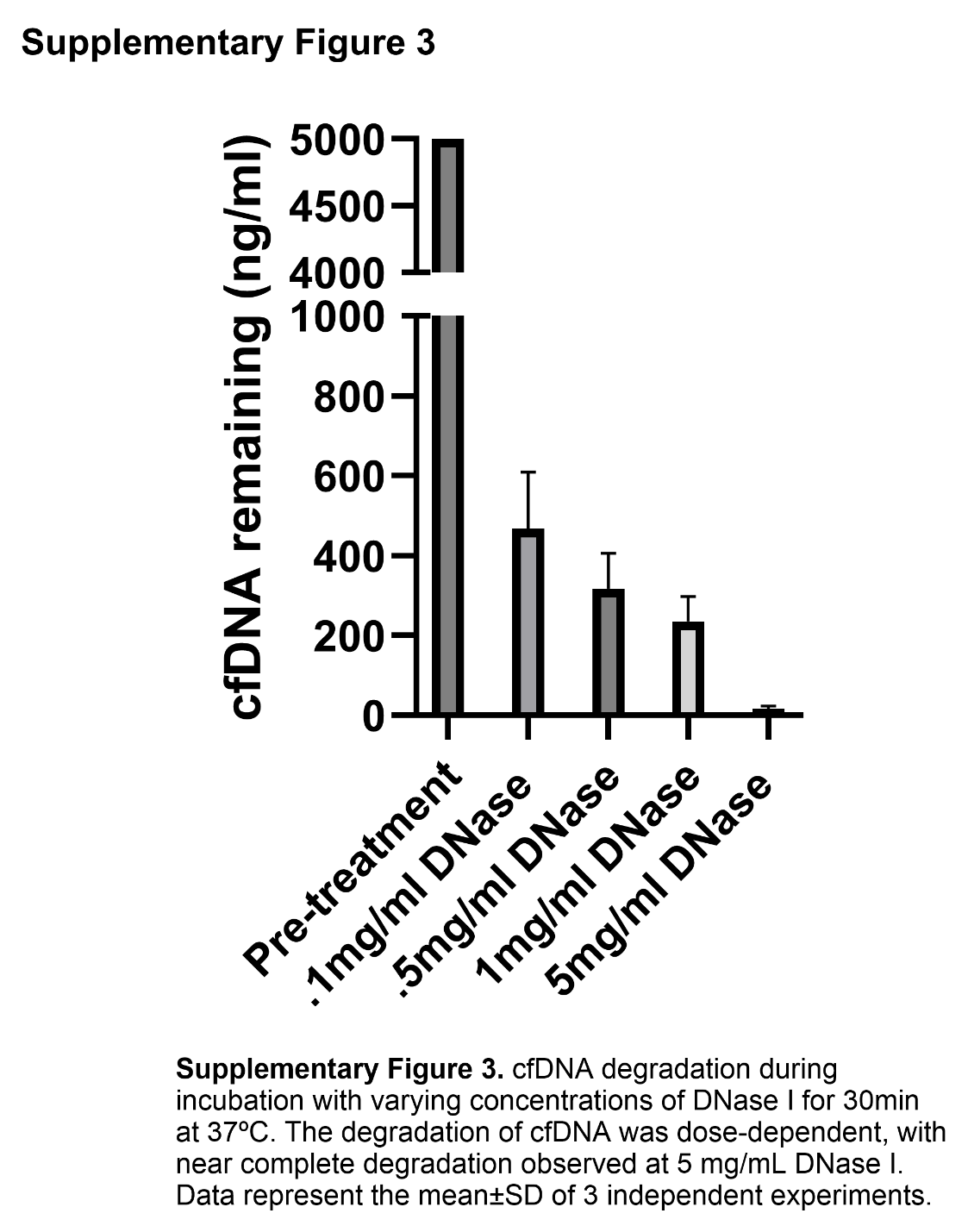

Supplement: Supplementary file 1 [file Supplementaryfile1.docx]
